# A New Approach for Assessment of Mental Architecture: Repeated Tagging

**DOI:** 10.1371/journal.pone.0029667

**Published:** 2012-01-09

**Authors:** Aire Raidvee, Agne Põlder, Jüri Allik

**Affiliations:** 1 Department of Psychology and Estonian Center of Behavioral and Health Sciences, University of Tartu, Tartu, Estonia; 2 Estonian Academy of Sciences, Tallinn, Estonia; CNRS - Université Claude Bernard Lyon 1, France

## Abstract

A new approach to the study of a relatively neglected property of mental architecture—whether and when the already-processed elements are separated from the to-be-processed elements—is proposed. The process of numerical proportion discrimination between two sets of elements defined either by color or by orientation can be described as sampling with or without replacement (characterized by binomial or hypergeometric probability distributions respectively) depending on the possibility to tag an element once or repeatedly. All empirical psychometric functions were approximated by a theoretical model showing that the ability to keep track of the already tagged elements is not an inflexible part of the mental architecture but rather an individually variable strategy which also depends on conspicuity of perceptual attributes. Strong evidence is provided that in a considerable number of trials, observers tagged the same element repeatedly which can only be done serially at two separate time moments.

## Introduction

The way mental processes are organized—their architecture—has been one of the main concerns for both psychologists and neuroscientists [cf. 1]. The question of whether people perform perceptual and mental operations in parallel or in series, has been pivotal in many of these pursuits [2], [3]. Overwhelmingly, the debate about serial *vs* parallel processing has been concentrated on reaction time data. In a seminal experiment, Sternberg [4] demonstrated that when observers judge whether a test symbol is contained in a short memorized sequence of symbols, their mean reaction-time increases linearly with the length of the sequence. The linearity and slope of the function were interpreted as strong evidence in favor of an internal serial-comparison process whose average rate is between 25 and 30 symbols per second. However, as it was soon shown by a thorough theoretical analysis, the distinction between serial and parallel processing is constrained by model mimicking: parallel models can lead to exactly the same predictions as serial ones despite the completely different psychological assumptions they are based on [3], [5].

One lesson that can be derived from the serial *vs* parallel controversy is that it cannot be resolved in isolation from other relevant attributes of the cognitive architecture. For example, it became evident that the questions about stopping rule – the conditions under which the system ceases processing and generates a response – or the questions about capacity limitations, are inevitably linked to the question about serial *vs* parallel architecture [3]. Considering this lesson, it is surprising that even though a number of studies exist on serial *vs* parallel processing in the context of enumeration accuracy of independent sets, e.g. [6], [7], the serial *vs* parallel debate has almost entirely escaped the numerosity discrimination accuracy problem. At least one study has shown similar counting and subitizing processes to those measured in standard enumeration tasks to be involved in the number discrimination task with a single stimulus set [8]. Yet, not much information is available about the nature of processes involved in numerosity discrimination in case the stimulus display contains multiple distinct sets.

In the following, we use the term *counting* as referring to any process aimed at finding the total number of elements in a set. The term is neutral with respect to the temporal properties of the processes involved: counting can be parallel, serial, or mixed.

It has long been known that it takes at least 5–6 years before children are able to learn all principles that are needed for counting, including assignment of numerals for objects [9]. But even after learning to count it is not guaranteed that perceptual mechanisms follow the principles used in verbal and propositional thinking. It is possible that even the most fundamental principle of numeration – the one-to-one correspondence between items and counting tags in the process of transformation of every item from the to-be-counted category to the already-counted category – cannot always be obeyed [cf. 9]. Perceptually it may be difficult to assign only one counting tag to every object with the purpose of preventing the same object from being counted twice. When the searched objects lack a clear structure it may be difficult to keep track of which object is already counted and which is still on the waiting list.

To the best of our knowledge, there is no generally accepted method for establishing whether or not the tagging process follows exactly the one-to-one principle. Unlike many previous studies which have used analysis of reaction times to differentiate between serial *vs* parallel processing styles, we attempt to reveal this property of mental architecture on the basis of probability distribution of responses. Our approach stems from an ideal observer analysis which purpose is to establish an absolute scale of performance for an ideal perceptual device that is limited only by stochastic characteristics of the stimulus itself [10]. Let's suppose that the observer's task is to discriminate the numbers of two distinct sets of randomly distributed elements. These two sets can be distinguished by their spatial position, occupy two separate areas, for example [11], or they can be intermixed but distinguished by a certain visual attribute, such as color or orientation [12]. This is a relatively simple task, as even pigeons, with a brain weighing less than 3 g, can be trained to discriminate numerical proportion in the mixtures of two types of elements with considerable accuracy [13], [14]. As expected, an ideal perceptual device can notice even one element difference irrespective of the total number of elements. Real observers, human or nonhuman, usually perform less accurately, presumably because their decisions seem to be based on only a fraction of available items. It is conceivable that instead of all presented elements the real observers are able to take into account only a fraction of the elements, especially when these elements have a random spatial distribution and are presented for a very short time. Formally, this situation resembles the inverse probability problem in which a sample of randomly selected elements serves as a basis for inference about the true proportion of elements hidden from the observer. Jacob Bernoulli in his posthumous *Ars conjectandi* (1713/1899) devised an ingenious urn problem as an idealized mental exercise in which some objects or concepts of real interest (such as people, event outcomes, visual objects, etc.) are represented as colored balls or pebbles which are drawn, one after another, randomly from the urn and their color is noted. Every probability textbook teaches that balls or pebbles once extracted can or cannot be returned to the urn, which leads to two distinct probability distributions for the number of balls of a given color: the binomial and hypergeometric distributions, respectively. These two different replacement schemes, however, have an important application to the problem of mental architecture. Provided that Bernoulli's urn model describes sufficiently accurately what happens in the perception of numerical differences, the scheme of sampling with replacement (leading to the binomial distribution) implies that there is no tagging of which elements are already counted and which are not: the same element can, in principle, be inspected more than once. Consequently, if empirically determined psychometric functions for numerical discriminations between two sets of items are better described by binomial than hypergeometric distribution, it would provide evidence that some of these elements are inspected twice or more times which, understandably, can only be done serially at two or more different time moments. On the other hand, the scheme of sampling without replacement (leading to the hypergeometric distribution) implies that there is accurate one-to-one tagging of which elements are already counted and which are not, leading to an element being inspected only once, maximally. The attribution of one-to-one counting tags (corresponding to the sampling scheme without replacement) is by itself neutral to the problem of parallel or serial counting.

If an observer strictly adhered to the hypergeometric model (see equations (3) and (4) in the Methods section) with the parameter *K* (the number of elements taken into account in the decision process) being equal to the total number of elements in the stimulus display, *N*, then he or she would always determine correctly which of the two types of the elements is more numerous. The fact that the real observers in our experiments make errors indicates, within the proposed approach, that either they only take into account proper subsets of the elements (adhering to the hypergeometric model with *K*<*N*) or they count some of the elements more than once, adhering, at least partially, to the binomial model. Our analysis below indicates that both these possibilities take place: to account for the data best we need to assume that the observers in some trials use the hypergeometric model and in other the binomial model, with *K* varying from trial to trial. In relation to the seriality vs parallelity of counting, the conformity of the data with the hypergeometric model (i.e., sampling without replacement, one-to-one tagging of selected elements) leaves the question of seriality vs parallelity open. But once the data are shown to require the binomial model for at least a fraction of all trials, one has to accept that some elements can sometimes be counted more than once, and this can only be done serially, at two or more separate time moments.

The overall aim of the experiments was to introduce a new approach for the assessment of mental architecture, namely the property of whether, in the process of proportion discrimination of multiple stimulus sets, certain elements were being counted repeatedly. In our view, the aim was achieved by showing that this is indeed the case at least in some of the trials.

## Methods

### Ethics Statement

The study has been approved by the local Research Ethics Committee.

**Figure 1:**
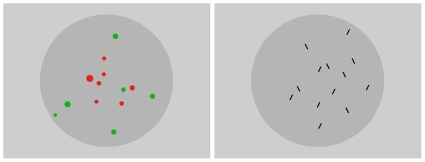
Stimulus configurations in the two experiments. Schematic view of stimulus configurations used in the numerosity discrimination experiment using color (left panel) or orientation (right panel) as a distinctive attribute.

Four 20-year-old female observers with normal or corrected to normal vision were asked to decide which of the two distinctive sets of objects were more numerous by pressing one of two buttons. In two separate series these two sets of objects were distinguished either by color or by orientation. A schematic view of the two types of stimulus configurations is shown in Figure 1. In the first series a randomly distributed collection of red and green circles was presented. The red and green circles had a luminance of about 23.5 cd/m^2^. To diminish the impact of total red *vs* green area on the responses, size of the circles was randomly varied in the range of 11 to 22 minutes of arc. In the second series of the experiments a collection of short black line segments of luminance 0.3 cd/m^2^ and tilt of 20° either to the left or to the right from the vertical direction was presented. The width and length of a line subtended 2′ and 19′ respectively (and height of its vertical projection 16′). Both types of stimuli were presented within an elliptical gray background with luminance of 54 cd/m^2^ and with lengths of horizontal and vertical axes 8.86° and 8.70° respectively. This elliptical background was in the center of a rectangular area of luminance 64 cd/m^2^ filling the rest of the screen. In order to avoid overlaps between elements, each element was positioned within an invisible inhibitory area which prevented other elements to be closer than 22′. Each stimulus element had a high contrast to guarantee its 100% identification would it have been presented in isolation. The total number of objects *N* presented on the display was kept constant through each experimental session and was equal either to *N* = 9 or 13 elements. These two relatively small values were chosen because the difference between the response probabilities from the binomial *vs* hypergeometric models is greater in case the total number of elements is small. During experimental sessions, the relative proportion of the type *A* and type *B* elements was varied. For example, for the total number of *N* = 9 the relative proportions of *A* (red or tilted to the left) and *B* (green or tilted to the right) element categories were the following: 1∶8, 2∶7, 3∶6, 4∶5, 5∶4, 6∶3, 7∶2, and 8∶1. The stimuli were presented at a viewing distance of 170 cm for 200 milliseconds, with 3 seconds for responding.

All stimuli were generated on the screen of a Mitsubishi Diamond Pro 2070SB 22″ color monitor (frame rate was 140 Hz with the resolution 1024×769 pixels) with the help of a *ViSaGe* (Cambridge Research Systems Ltd.) stimulus generator. Every stimulus condition was replicated 100 times. Choice probability of the red circles was plotted as a function of the proportion of red elements *N*_R_ in the total number of elements on the display *N = N*_R_+*N*_G_. Similarly in the orientation experiment, probability of the choice of the leftward tilted elements was measured as a function of the proportion of leftward tilted elements *N*_(\)_ in the total number of elements on the display *N = N*_(\)_+*N*_(/)_.

### Mathematical expression of the psychometric models

The probabilities of a certain choice response for odd and even *K* from the binomial model are given by equations (1) and (2):
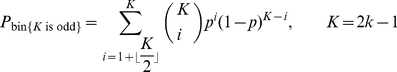
(1)

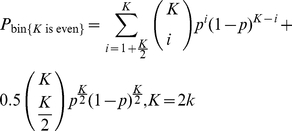
(2)where

*k* is any positive natural number;
*p* is the proportion of a certain type of elements to the total number of elements (either *N*_A_/(*N*_A_+*N*_B_) or *N*_B_/(*N*_A_+*N*_B_), depending on the experimental definition;
*K* is the number of elements taken into account in the decision process.

The probabilities of a certain choice response for odd and even *K* from the hypergeometric model are given by equations (3) and (4):
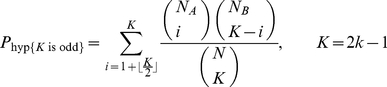
(3)

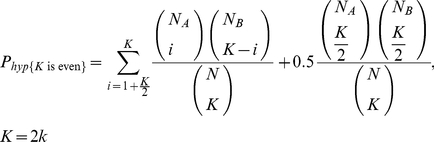
(4)where

*k* is any positive natural number;
*N*_A_ is the number of type *A* elements in the stimulus;
*N*_B_ is the number of type *B* elements in the stimulus;
*N* is the total number of elements in the stimulus (*N* = *N*_A_+*N*_B_);
*K* is the number of elements taken into account in the decision process.

As stated above, one only needs to consider either odd or even values of *K* because the probabilities given by a pair of equations (either those for the binomial model or for the hypergeometric model) are equal, given equal values for *k*.

## Results

**Figure 2:**
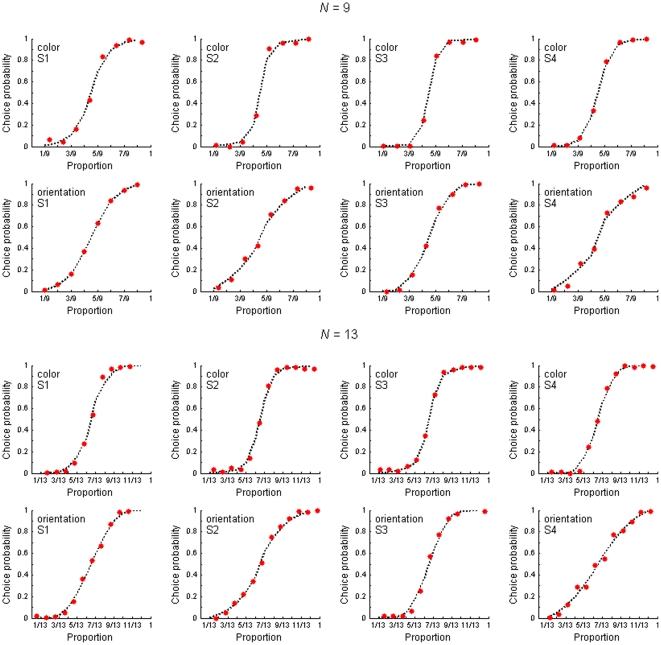
The best fitting theoretical models (dotted line) *vs* empirical results (red points). The choice probability as a function of the proportion of the chosen response category for four observers, two discrimination tasks (color and orientation), and two numbers of elements (*N* = 9 and 13). Each point is a probability estimate computed from 100 trials. The dotted line represents the best fitting theoretical mixture model shown in Tables 1.A and 2.A.

The obtained psychometric functions are shown in Figure 2. The probability of the choice of “red” (color experiment) or “leftward tilt” (orientation experiment) are plotted as a function of the proportion of the respective type of elements in the total number of displayed elements. As expected, the choice probability monotonically increases with the increase in the proportion of the indicated elements.

**Figure 3:**
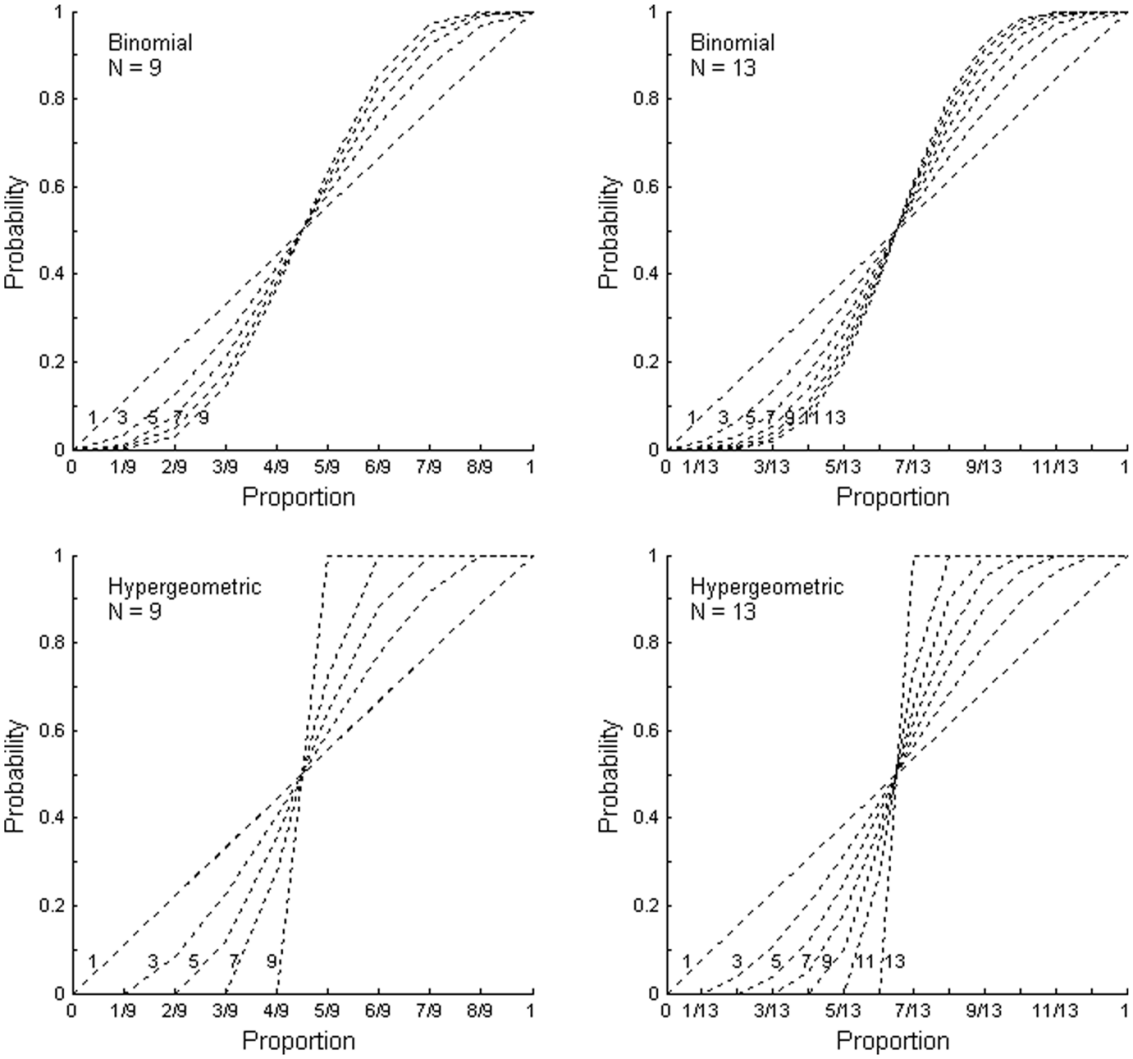
All possible theoretical models. All possible theoretical models corresponding to binomial (bin*_K_*) or hypergeometric (hyp*_K_*) distributions with the length of trials *K*.

It is assumed that the observer's decisions between response categories *A* and *B* are based on the inspection of *K* elements that are randomly selected from all available elements *N*. If the number of the *A*-type elements *K*_A_ in the selection exceeds the number of *B*-type elements (*K*_A_>*K*_B_), then the response category “*A*” is chosen; in the opposite case the response category “*B*” is chosen. If the numbers of *A* and *B* elements happen to be equal (*K*_A_ = *K*_B_) for an even number of selected elements *K*, then the choice between “*A*” and “*B*” response categories is random with probability 0.5. Following this simple decision rule it is easy to compute all theoretical cumulative probability functions for binomial and hypergeometric distributions. Figure 3 demonstrates these theoretical binomial and hypergeometric models for odd numbers of selected elements *K* (the sample size). One only needs to consider odd numbers of elements since *K* = 2*k*−1 (odd) and *K* = 2*k* (even) yield identical predictions. The equivalence of *K* = 2*k*−1 and *K* = 2*k* is easy to demonstrate numerically for any arbitrary *k* value or demonstrate their formal equivalence by using, for example, Wolfram's *Mathematica*. However, an analytic proof seems to go beyond ordinary algebra. The mathematical formulations of response probabilities from both types of models – binomial and hypergeometric – are given in the Methods section.

Only in a few cases were the empirical psychometric functions close enough to one of these model predictions. This outcome is expected since it would be unrealistic to assume that the observer can use a fixed number of elements *K* in each trial through the whole sequence of trials. It is more realistic to assume that the number of selected elements *K* is a variable and changes from one trial to another. Also, there is no clear reason to hold any one specific combination of theoretical models strictly superior to the others as, within error limits, many mixture models are able to provide a comparable fit. Therefore, the emphasis of the current analysis is to estimate the relative performance of the hypergeometric models to that of a combination of both hypergeometric and binomial models. We are greatly indebted to Ehtibar Dzhafarov for suggesting the described approach. At the heart of the underlying logic lies the assumption that in case any binomial component(s) is/are able to improve the overall fit of the mixture model (with the maximum number of possible mixture components held equal to the number of respectively possible hypergeometric models) then that would be an indication in support of serial processing in at least some of the trials.

An approximation algorithm based on least squares optimization was written which looked for the weighted combination of all theoretical models which minimizes the sum of squared errors between theoretical predictions and points of empirical functions. Prior to plotting the best mixture of theoretical models *vs* the empirical psychometric functions, the latter were shifted to the left or right to make their mean (μ) equal to 0.5. If the mean of all responses deviates from the expected 0.5 then it characterizes a response bias towards one of the two response alternatives. As expected, the empirical means were close to 0.5, ranging from 0.44 to 0.53.

The best predictions of the mixtures of theoretical models are shown in Figure 2 as continuous psychometric functions. The parameters of these best fitting mixture models are shown in Tables 1.A and 2.A. The number in the column corresponding to the theoretical model (bin*_K_* or hyp*_K_*) indicates the percentage of trials in which each of these models is expected to be used. For example, in the first row in Table 1.A the mixture model is described as **31**•hyp_5_+**26**•hyp_7_+**15**•hyp_9_+2**8**•bin_3_, which means that for the observer S1 the best fit was obtained when the hypergeometric model with the sample size of either *K* = 5, *K* = 7 or *K* = 9 was supposed to be used in 31%, 26% and 15% of all the individual trials, respectively, and the binomial model with the sample size of *K* = 3 was used in the remaining 28% of the trials. Even a visual inspection can reveal that the fit to all 16 empirical psychometric functions shown in Figure 2 was excellent. This was confirmed by more formal tests showing that the predicted psychometric functions were able to explain on average 98.86% of the total response variance. Thus, only about 1.14% of total variance on average remained unexplained and could be attributed to measurement error.

**Table 1:** 

A. The combinations of theoretical hypergeometric and binomial models providing the best fit to the empirical psychometric functions (*N* = 9).									
Observer	hyp_3_	hyp_5_	hyp_7_	hyp_9_	bin_3_	bin_5_	bin_7_	bin_9_	%Error
**COLOR (** ***N*** ** = 9)**									
**S1**		31	26	15	28				1.5677
**S2**			45	39	15			1	1.0888
**S3**			61	32	7				0.2616
**S4**		29	48	15	8				0.3019
**ORIENTATION (** ***N*** ** = 9)**									
**S1**			23		9	60		8	0.0005
**S2**		10		12	77	1			0.8859
**S3**	16	73		11					0.9085
**S4**	18			19	63				1.2777

B. The combinations of theoretical hypergeometric models providing the best fit to the empirical psychometric functions (*N* = 9).										
	COLOR					ORIENTATION				
Observer	hyp_3_	hyp_5_	hyp_7_	hyp_9_	%Expl	hyp_3_	hyp_5_	hyp_7_	hyp_9_	%Error
**S1**	30	36	18	16	1.5958	71		29		0.0095
**S2**	18		42	40	1.0914	91			9	0.9217
**S3**	8		60	32	0.2647	16	73		11	0.9085
**S4**	12	23	50	15	0.3034	85			15	1.3358

*Note*: *N* = number of elements on the display; %Error = the percentage of variance unexplained by the mixture of the theoretical models; bin*_K_* = the binomial model sampling *K* elements; hyp*_K_* = the hypergeometric model sampling *K* elements.

**Table 2:** 

A. The combinations of theoretical hypergeometric and binomial models providing the best fit to the empirical psychometric functions (*N* = 13).													
Observer	hyp_3_	hyp_5_	hyp_7_	hyp_9_	hyp_11_	hyp_13_	bin_3_	bin_5_	bin_7_	bin_9_	bin_11_	bin_13_	%Error
**COLOR (** ***N*** ** = 13)**													
**S1**	13	2	54	7	11	13							3.3804
**S2**				41	43		16						1.5035
**S3**			5	12	58	4	21						0.1676
**S4**			65	29		6							1.5758
**ORIENTATION (** ***N*** ** = 13)**													
**S1**		72	28										0.7928
**S2**	47		17		8	8	20						1.3801
**S3**			74	20			6						2.1331
**S4**	61				5		34						1.0062

B. The combinations of theoretical hypergeometric models providing the best fit to the empirical psychometric functions (*N* = 13).														
	COLOR							ORIENTATION						
Observer	hyp_3_	hyp_5_	hyp_7_	hyp_9_	hyp_11_	hyp_13_	%Expl	hyp_3_	hyp_5_	hyp_7_	hyp_9_	hyp_11_	hyp_13_	%Error
**S1**	13	2	54	7	11	13	3.3804		72	28				0.7928
**S2**	17			40	43		1.5111	72		9	2	9	8	1.3822
**S3**	24		3	10	59	4	0.1789	6		76	18			2.1339
**S4**			65	29		6	1.5758	98				2		1.0200

*Note*: *N* = number of elements on the display; %Error = the percentage of variance unexplained by the mixture of the theoretical models; bin*_K_* = the binomial model sampling *K* elements; hyp*_K_* = the hypergeometric model sampling *K* elements.

The maximum number of components in the best fitting mixture models is four in case *N* = 9 (Table 1.A) and six in case *N* = 13 (Table 2.A) in order to keep the number of regressors equal to that of the competing mixture composed of hypergeometric models only. The best predictions obtained by hypergeometric models alone are given in Tables 1.B and 2.B. In most cases does the fit of the mixture containing binomial model(s) surpass that of the respective mixture containing only hypergeometric models. In Tables 1.A and 2.A, in cases where the binomial component improved the fit, the number presenting the proportion of unexplained variance is underlined. Since in 12 out of 16 cases addition of the binomial component improved the fit one can conclude that there were a significant number of trials in which the observers were not able to track exactly the elements that were already counted and those that were not.

In general, it is known that numerical discrimination based on color is more efficient than one based on geometric attributes, such as orientation [cf. 12]. This seems to be in agreement with our results: across all conditions and observers on average 5 elements were taken into account in orientation discrimination task and 7.5 elements when color was the distinguishing attribute.

In both types of tasks the hypergeometric distribution provided a better fit than the binomial one: in 65.3% of all trials when applied to discrimination on the basis of orientation, and in 88% of trials when applied to discrimination based on color. It was not entirely surprising to discover some small individual differences since it was previously shown that some participants adhered to a serial processing profile in most conditions while other participants could exhibit parallel-like strategy in some conditions at least [15].

## Discussion

In order to enumerate objects accurately it is necessary to follow certain rules. One of these basic rules is the maintenance of the one-to-one relationship between objects and tags assigned to these objects: every object needs to be tagged only once. It is generally unknown whether and how well different perceptual processes are able to separate the to-be-counted items from the already-counted ones. In this study we have proposed a new approach to this problem. Although the question of whether and when people can perform perceptual and mental operations in parallel or in series has been dominating debates about mental architectures, it was also made clear that this central question can be answered only when other related questions such as stopping rules, selective influence [16], [17], and capacity limitations have been answered as well [1], [18]. The one-to-one principle of tagging obviously belongs to the same category of the related problems. In this study we presented strong evidence that it is reasonable to assume that in a considerable number of trials observers behave as if they are not able to keep track of the elements they have already counted. It is very likely that when forming their decision, they have taken the same element into account repeatedly. Since the same element can be visited twice or more times only on different time moments, this is a strong indication that at least some operations are executed serially.

The obtained evidence does not allow to assert that the adherence to the one-to-one tagging principle is an inflexible part of the mental architecture. Previous studies have shown that depending on the observer and stimulus conditions the parallel processing strategy can be used in some and the serial processing strategy in other situations [15]. Our results seem to suggest that in perceptual tasks that can be solved more automatically and spontaneously, like discriminations based on color, the observers have a tendency to keep track of elements that have already been counted. By contrast, in tasks like discrimination based on orientation that require more deliberation and scrutinizing of each element, the observers tend to confuse which elements have already been counted and which have not. Although the accurate tagging of the counted elements does not necessarily mean that the processing is executed in parallel, lack of the one-to-one tagging implies that at least some elements are processed serially, one after another. However, these are not inflexible rules. For instance, one of the four observers performed better in the orientation based discrimination task than in the color discrimination task. This seems to suggest that avoidance of repeated tagging of elements is not a rigid part of mental architecture but rather a flexible strategy that can be changed and, if necessary, learned. This conclusion is supported by the fact that no single theoretical model was able to provide a satisfactory explanation for most of the empirical psychometric functions. The best fit was found when predictions of different theoretical models were combined. This implies that the observers do not adhere to only one strategy even during one experimental session. We can only guess the number of different strategies used during one session but at least three appear to be the norm in most cases.

The observed individual differences are particularly interesting in the light of a recent report showing that the ability to discriminate numbers of elements in two sets was correlated with a psychometrically measured intelligence [19]. It is an intriguing possibility that the ability to keep track of elements which have already been counted (together with the sample size one is able to base his/her decisions upon), forms a precondition for numerical intelligence which, in turn, among other faculties, gives rise to general intellectual abilities.
